# Patient reported physical and mental health changes associated with a comprehensive cardiovascular risk reduction program for women with breast cancer receiving potentially cardiotoxic chemotherapy

**DOI:** 10.1186/s40959-021-00107-w

**Published:** 2021-05-31

**Authors:** Michael G. Fradley, Mohammed Alomar, Marcus W. Kilpatrick, Bernadette Shields, Nhi Tran, Amey Best, Erika Bianco, Merna Armanious, R. Ashton Vautier, Kevin Kip, Theresa M. Beckie, Roohi Ismail-Khan

**Affiliations:** 1 Cardio-Oncology Center of Excellence, Division of Cardiology, Department of Medicine, University of Pennsylvania, 3400 Civic Center Blvd, Philadelphia, PA 19104 USA; 2 Cardio-Oncology Program, Moffitt Cancer Center, University of South Florida College of Medicine, Tampa, USA; 3 Exercise Science Program, College of Arts and Sciences, University of South Florida, Tampa, USA; 4 Department of Clinical Analytics, Health Sciences Division, University of Pittsburgh, Pittsburgh, USA; 5 College of Nursing, University of South Florida, Tampa, USA

**Keywords:** Cardio-oncology, Cardiotoxicity, Breast cancer, Cardiovascular, Behavioral health

## Abstract

**Objective:**

Women with breast cancer (BCA) and cardiovascular disease (CVD) risk factors are at increased risk of developing cardiovascular complications when exposed to potentially cardiotoxic cancer therapy. The benefit of aggressive CVD risk factor modification to reduce adverse treatment-related psychologic and biologic effects is not well established.

**Methods:**

Using a single group pre-test, post-test design, 33 women with BCA receiving anthracycline and/or trastuzumab therapy participated in a 6-month comprehensive CVD risk reduction program involving formal cardio-oncology evaluation along with regular motivational counseling for improved nutrition and physical activity. Study parameters were assessed at baseline and 6 months with paired t-tests used to evaluate changes after the intervention.

**Results:**

The mental component summary score assessed by SF-36_V2_ improved significantly after program completion (45.0 to 48.8, effect size 0.37, *p* = 0.017), however the physical component summary score declined (46.2 to 40.9, effect size − 0.53, *p* = 0.004). Despite this decline in perceived physical health, markers of health-related fitness and nutritional status were maintained or improved. Systolic and diastolic blood pressure also improved after the intervention (136.7 to 124.1 mmHg, *p* = 0.001 and 84.0 to 78.7 mmHg, *p* = 0.031, respectively). No significant change in resting heart rate, body mass index, lipids, hemoglobin A1C, or left ventricular ejection fraction was observed.

**Conclusions:**

Patient-reported mental health improved significantly in women with BCA enrolled in a comprehensive CVD risk reduction program despite exposure to potentially cardiotoxic therapies. This study provides preliminary data for future randomized controlled trials evaluating the effects CVD risk reduction program in high-risk breast cancer cohorts.

## Introduction

Breast cancer is the most common malignancy in women, accounting for 15% of new cancer cases, and almost 7% of cancer-related deaths [1]. Nevertheless, heart disease remains the leading cause of mortality in women at 22%, yet only 56% of women are aware of this issue [2]. In particular, postmenopausal women with breast cancer have higher cardiovascular disease (CVD) mortality than women without breast cancer; a risk that manifests around 7 years after cancer diagnosis [3]. Moreover, women with breast cancer (BCA) are at increased risk of developing CVD complications when exposed to treatment regimens consisting of anthracyclines, HER2 targeted agents such as trastuzumab, and/or left sided breast radiation [4]. The likelihood of developing these cardiotoxicities are increased in women with baseline CVD or risk factors such as obesity, hypertension, or diabetes [4, 5].

In 2018, the American Heart Association (AHA) published its first scientific statement on CVD and breast cancer in an effort to increase awareness of the problem and highlight the need for proper CV prevention in this patient population [6]. Aggressive CVD risk factors modification is recommended for patients with cancer [5], however, the effects of risk factor modification on CVD outcomes in cancer patients remains an area of active investigation. In addition, to traditional medical interventions, there is increasing attention on nutrition and exercise to mitigate CVD risk in cancer patients and survivors [7]. There is a paucity of data evaluating patient-reported quality of life outcomes in this unique population. Our study aimed to prospectively examine the psychosocial and physical benefits of a structured program for CVD risk monitoring and modification for high risk breast cancer patients receiving potentially cardiotoxic cancer therapy.

## Methods

### Study setting

All procedures involving human participants were in accordance with the ethical standards of the institutional and/or national research committee and with the 1964 Declaration of Helsinki and its late amendments or comparable ethical standards. The study was approved by the University of South Florida Institutional Review Board and the H. Lee Moffitt Cancer Center (MCC) and Research Institute Scientific Review Committee (Pro00023536; MCC #18344). All subjects provided written informed consent prior to enrollment.

This prospective study used a single group, pre-test, post-test design. Women with a new diagnosis of breast cancer scheduled to begin treatment with either anthracycline-based chemotherapy (ABT) and/or HER2 targeted therapy with at least one baseline CVD risk factor comprised the study population. The study was conducted at MCC and participants were recruited from the Women’s Oncology and Senior Adult Oncology programs. Women were included in the study if they met all of the following inclusion criteria: age 40–79 years; at least one baseline CVD risk factor including hypertension, hyperlipidemia, diabetes, active smoking, or obesity (BMI ≥30 kg/m^2^); treatment with ABT and/or HER2 targeted therapy. Exclusion criteria included: lack of CVD risk factors listed above; history of heart failure/cardiomyopathy; evidence of vascular disease; prior myocardial infarction, percutaneous coronary intervention or coronary bypass surgery; prior heart valve surgery; use of non ABT or HER2 therapy; inability to exercise; inability to provide informed consent/significant cognitive impairment; metastatic breast cancer; male gender; lack of telephone access; lack of English fluency.

### Cardio-oncology risk reduction program

**Fig. 1:**
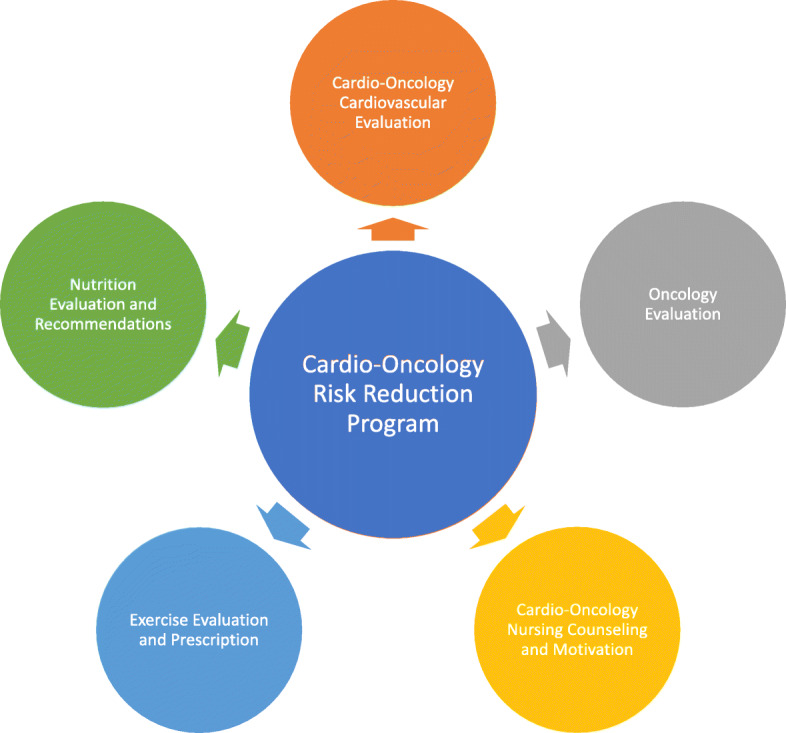
Cardio-Oncology Risk Reduction Program Structure

A multi-disciplinary cardio-oncology risk reduction program was developed, incorporating cardio-oncology medical evaluation, nutritional counseling, exercise evaluation and recommendations, and nursing communication and motivational interviewing counseling (Fig. 1). Patients who met inclusion criteria and signed informed consent were referred to the cardio-oncologist involved in the clinical cardio-oncology program. Baseline physical and laboratory measures (listed below) were obtained and individualized cardiovascular medical care plans were determined as per standard of care with repeat evaluations at 3 and 6 months unless otherwise indicated. The cardio-oncologist also ensured participants were safe to undergo exercise. The patients were then evaluated by the exercise physiologist at baseline and 6-months and were provided with individualized aerobic, body weight callisthenic, and resistance band exercise prescriptions. They were also seen one time prior to starting treatment by a cancer nutritionist who provided nutrition guidance in the setting of cardiovascular disease and breast cancer. All baseline assessments occurred within 2 weeks prior to the initiation of treatment. Every 3 weeks after starting cancer treatment, participants were asked to provide a 24-h exercise and dietary log to improve participant accountability. Surveys were collected in bulk at scheduled follow up appointments. The participants were contacted by the nurse coordinator at 3 week intervals as a reminder to complete the logs and this also served as an opportunity to answer questions, provide psychosocial support and motivation, and address any barriers to exercise and healthy dietary habits. A motivational interviewing counseling style was used throughout the phone calls. The nurse coordinator expressed empathy, elicited the patients motivation for change, explored ambivalence about behavior change, supported the patients self-efficacy for change, and rolled with any resistance as appropriate. A manual of was used to maintain fidelity to the intervention.

### Outcome measures

Outcome measures were obtained at the time of enrollment and again at the 6-month follow up cardio-oncology clinic visit. A complete physical exam was performed including vital signs (heart rate and blood pressure), body weight, and height measurements. Heart rate and blood pressure were measured using an automated sphygmomanometer using the standard American Heart Association Protocol [8]. Lipid panel (total cholesterol, triglycerides, high density lipoprotein [HDL] cholesterol, low density lipoprotein [LDL]) cholesterol and hemoglobin (Hgb) A1C were measured from a fasting blood sample using standard technology in the MCC core laboratory. An echocardiogram was also performed using Phillips Epiq® echocardiography equipment as part of the standard-of-care cancer treatment regimens. Typical echocardiographic parameters including ejection fraction (EF) using the Simpson’s method were recorded. Quality of life was assessed using the Short Form Health Survey (SF-36_V2_) which is comprised of 36 questions about 8 dimensions of health including vitality, physical functioning, bodily pain, general health perceptions, physical role functioning, emotional role functioning, social role functioning and mental health. Scores range from 0 to 100 with higher scores representing better perceived health [9]. Dietary practices were assessed using the Rapid Eating Assessment for Participants, Short Version (REAP-S) survey. This survey includes 13 questions each scored 1–3. The maximum score on the survey is 39, with higher scores indicating higher diet quality [10].. Assessment of aerobic fitness using a submaximal treadmill test and muscular fitness using hand weights was completed by the study exercise physiologist at baseline and again at 6 months.

### Statistical analyses

Descriptive statistics were used to describe all study variables and participant characteristics. SF-36 subscale scores and measures of cardiovascular risk factors, fitness and dietary health were all scored as continuous variables, with the SF-36 data scored using the original (Ware) algorithm. Therefore, paired t-tests were used to evaluate pre-to-post changes in these continuous variables from baseline to after the intervention. In addition, standardized effect sizes (from pre-to-post outcome scores) were calculated using the within-person single group pretest–posttest design described by Morris and DeShon [11]. Statistical significance was defined as a *P* value of < 0.05.

## Results

A total of 40 women with BCA receiving anthracyclines and/or trastuzumab were consented for study with 33 women completing the program. Seven subjects withdrew from the study prior to the first cardio-oncology clinic all whom cited physical and/or emotional limitations. Baseline patient demographics are shown in Table 1. The mean age of participants was 51 years with a standard deviation of 12, 52% of participants were white, non-Hispanic, and 82% received anthracycline regimens. Hypertension was the most common baseline CVD risk factor (55%).

**Table 1:** Baseline Characteristics of Study Population

Baseline Characteristics of Study Population (***n*** = 33)	
Age – years	51 ± 12
Race – no (%)	
White, non-Hispanic	17 (52)
Black	11 (33)
Hispanic	4 (12)
Asian	1 (3)
Anthracycline use – no (%)	27 (82)
HER2 Blockade use – no (%)	6 (18)
Body Mass Index^a^	30 ± 10
Hypertension – no (%)	18 (55)
Hyperlipidemia – no (%)	9 (27)
Diabetes – no (%)	6 (18)
Active Smoking – no (%)	3 (9)

Values are means ± standard deviation unless otherwise noted

^a^Body Mass Index: weight in kilograms divided by the square of the height in meters

Patient reported quality of life outcomes using the SF-36_v2_ health survey before and after participating in the cardio-oncology risk reduction program are shown in Table 2. The only SF-36 subscale score to demonstrate significant change over the course of the study was mental health (44.3 to 49.4, effect size 0.47, *p* = 0.004). Overall,the mental component summary score (MCS) significantly improved after program completion (45.0 to 48.8, effect size 0.37, *p* = 0.017) however the physical component summary score (PCS) significantly declined (46.2 to 40.9, effect size − 0.58, *p* = 0.004)..

**Table 2:** SF-36 Health Survey Scores Before and After the Cardio-Oncology Risk Reduction Program

SF-36 Sub-Scale Measures	Baseline	Follow-up	Difference	Effect Size	***P***-value
Physical Functioning	47.7 ± 9.8	44.3 ± 12.3	−3.4 ± 9.0	−0.38	0.07
Physical Role	42.6 ± 12.5	39.9 ± 13.9	−2.7 ± 14.3	−0.19	0.30
Bodily Pain	45.2 ± 9.5	44.3 ± 11.6	−0.8 ± 12.1	−0.07	0.48
General Health Perception	44.9 ± 10.1	42.6 ± 10.5	−2.3 ± 11.1	−0.21	0.26
Mental Health	44.3 ± 13.3	49.4 ± 11.6	5.1 ± 10.8	0.47	**0.004**
Emotional Role Limitations	45.6 ± 13.4	47.8 ± 12.6	2.2 ± 11.5	0.19	0.21
Social Functioning	44.9 ± 13.1	42.6 ± 12.6	−2.3 ± 13.4	−0.17	0.36
Vitality/Energy	47.3 ± 11.8	44.8 ± 13.4	−2.5 ± 11.2	−0.22	0.23
Physical Component Summary (PCS)	46.2 ± 9.9	40.9 ± 11.9	−5.4 ± 10.2	−0.53	**0.005**
Mental Component Summary (MCS)	45.0 ± 14.0	48.8 ± 11.5	3.8 ± 10.4	0.37	**0.02**

Values listed as means ± standard deviation; Bold indicates statistical significance result

Despite the decline in perceived physical health from the SF-36 health survey, markers of health-related fitness and nutritional status were maintained or improved over the course of the study (Table 3). Specifically, systolic and diastolic blood pressure significantly improved after the intervention (136.7 to 124.1 mmHg, *p* = 0.001 and 84.0 to 78.7 mmHg, *p* = 0.031 respectively). There was no significant change in resting heart rate, body mass index, lipids or Hgb A1C. Healthy eating choices improved as demonstrated by an increase in the REAP score (26.6 to 28.7, *p* = 0.007) as well as measures of physical fitness including the arm curl test (18.5 to 21.8, *p* = 0.008) (Table 3).

**Table 3:** Cardiovascular, Fitness and Dietary Health Parameters Before and After the Cardio-Oncology Risk Reduction Program

Variable	Baseline	Follow-up	***P*** Value
**Cardiovascular Clinical Evaluation**			
Systolic BP/Diastolic BP (mm Hg)	136.7 ± 19.4	124.1 ± 14.0	**0.001**
Diastolic Blood Pressure (mm Hg)	84 ± 12.9	78.7 ± 8.2	**0.03**
Resting Heart Rate (beats per minute)	81 ± 12.9	86 ± 13.5	0.19
Body Mass Index (kg/m^2^)	29.8 ± 9.8	31.5 ± 7.3	0.58
Body Fat (%)	41.1 ± 5.8	41.3 ± 3.8	0.66
**Cardiovascular Laboratory Evaluation**			
Total Cholesterol (mg/dL)	183.1 ± 43.6	184.6 ± 45.0	0.40
HDL Cholesterol (mg/dL)	55.1 ± 16.6	53.7 ± 14.7	0.34
LDL Cholesterol (mg/dL)	100.6 ± 40.1	104.4 ± 44.9	0.20
Triglycerides (mg/dL)	137.9 ± 71.6	155.4 ± 105	0.12
Hemoglobin A1C (%)	7.4 ± 8.3	5.6 ± 0.8	0.11
**Physical Fitness Evaluation**			
Arm Curl Test (repetitions)	18.5 ± 4.5	21.8 ± 4.7	**0.008**
Sit to Stand Test (repetitions)	12.8 ± 2.6	14.5 ± 2.9	0.05
Trunk Flexion Test (repetitions)	13.6 ± 4.1	12.6 ± 3.2	0.80
**Nutrition Evaluation**			
REAP Score	26.6 ± 4.7	28.7 ± 4.7	**0.007**

Values listed as means ± standard deviation; Bold indicates statistical significance; *BP* blood pressure, *REAP* Rapid Eating Assessment for Participants

Among the cohort of patients that had an echocardiogram (*N* = 30), there was no significant change in standard echocardiographic parameters including ejection fraction, left ventricular internal diameter in diastole or left atrial diameter (Table 4) after exposure to potentially cardiotoxic cancer therapy and participation in the cardio-oncology program.

**Table 4:** Echocardiographic Parameters Before and After the Cardio-Oncology Risk Reduction Program

Variable	Baseline	Follow Up	***P*** Value
Ejection Fraction (%)	62.1 ± 4.4	60.3 ± 4.7	0.11
Left Ventricular Internal Dimension, Diastole (mm)	40.9 ± 11.7	43.1 ± 6.0	0.78
Left Ventricular Septal Thickness (mm)	9.0 ± 3.0	9.5 ± 1.5	0.52
Left Ventricular Posterior Wall Thickness (mm)	10.9 ± 9.8	10.3 ± 1.5	0.10
Left Atrial Diameter (mm)	34.2 ± 14.9	30.8 ± 9.0	0.33

Values listed as means ± standard deviation; Bold indicates statistical significance

## Discussion

In this prospective study with a single group pre-test post-test design, a 6-month comprehensive CVD risk reduction program involving structured cardio-oncology evaluation with regular motivational interviewing counseling for improved nutrition and adherence to individualized physical fitness prescriptions resulted in improvement in patient-reported mental health despite breast cancer diagnosis and exposure to potentially cardiotoxic cancer treatments. Although patient-reported physical health declined, numerous measures of health-related fitness, dietary habits, and CV health were maintained or improved.

Although breast cancer and CVD remain common causes of morbidity and mortality in the United States, survival has improved significantly for both diseases due to improved screening and treatment [12]. There is also increasing recognition that both CVD and breast cancer share common risk factors, such as age, diet, and family history [6]. Around 80% of CVD can be prevented with interventions such as healthy diet, tobacco cessation, blood pressure and diabetes mellitus control and physical activity [13]. Similar interventions can also improve breast cancer outcomes yet there are no standard recommendations for the management and monitoring of CVD in cancer patients and survivors [14].

While much of the attention from cardio-oncology interventions has focused on the prevention of disease development such as heart failure or ischemic heart disease, there is an increasing body of literature demonstrating the positive impact of patient reported psychosocial outcomes on the long-term health of breast cancer patients [15, 16]. To assess the mental well-being of our patients, we used the mental component of the well-validated SF-36 [17]. The mental component score of the survey improved in our patients after the intervention, highlighting the potential psychological impact of a multidisciplinary approach to improving the cardiovascular health of breast cancer patients.

Despite the patient-reported improvement in mental well-being, participants in our study reported lower perceived physical health post-intervention. Nevertheless, objective markers of health-related fitness were maintained or improved over the course of the study. For example, both systolic and diastolic blood pressure significantly improved after the intervention. Systolic blood pressure reduced by 12 points, while diastolic blood pressure reduced by 6 points. This decline in blood pressure is similar to what has been reported with certain heart-healthy diets, such as the DASH (Dietary Approach to Stop Hypertension) eating plan [18, 19]. Similarly, we observed a statistically significant improvement in muscular endurance as assessed by arm curl repetitions after the intervention. The arm-curl test is a common and validated field test to assess for upper extremity strength via repetitions of elbow flexion and extension performed with a dumbbell over 30 s [20]. We hypothesize that this decline in the patient-reported perception of physical health is related to the challenges faced with cancer treatments such as chemotherapy, radiotherapy, and breast surgery. It is possible that this decline may have been more pronounced in a control group and the intervention in this study may have attenuated the overall decline. It is also important to recognize that statistically significant changes in QOL may not translate into clinically meaningful changes however. In general, a 5% change has been considered clinically meaningful in cancer patients. As such, the 5-point decrease in the PCS is likely to be clinically meaningful while the 3-point increase in the MCS may not have as much clinical significance.

It is important to motivate cancer patients to maintain physical activity during and after treatment. Women in their 40s with breast cancer have a mean cardiorespiratory fitness level 30–32% lower than age-matched controls [21]. Exercise training is the primary modality used to improved cardiorespiratory fitness within an increasing body of literature demonstrating its benefit in the breast cancer population. For example, a meta-analysis of 27 studies demonstrated a significant improvement in cardiorespiratory fitness after adjuvant therapy among women actively engaged in exercise training [22].

This is also one of a relatively small number of studies to objectively evaluate changes in CV nutrition habits in breast cancer patients. We utilized The Rapid Eating and Activity Assessment for Patients (REAP), a brief validated questionnaire designed to evaluate dietary and physical activity patterns with higher scores indicating healthier eating behaviors [10]. The REAP scores significantly increased indicating improved dietary habits as a result of our motivational interventions. This is consistent with recommendations from the American Heart Association to tailor nutritional counseling to the unique needs of cancer patients [7].

Our findings underscore the results from other studies that have aimed to identify non-pharmacological interventions to decrease CVD events in breast cancer patients. Jones and colleagues found exercise was associated with substantial reductions in the incidence of cardiovascular events in women with nonmetastatic breast cancer [23]. Similarly a recent retrospective study by Okwuosa and colleagues demonstrated that exercise exposure prior to breast cancer diagnosis was associated with a significant reduction in CVD events in long-term survivors [24]. Although our study was not designed to assess long-term CVD outcomes, the impact on quality of life and markers of health-related fitness may translate into improved and enduring health effects.

## Limitations

Our study has several limitations. The lack of a control group makes intervention effects harder to assess. The small sample size may decrease the power of this study to detect a difference in outcomes after the intervention and a larger study may yield different results. The rigorous patient follow-up may not be logistically feasible in all institutions outside of a clinical trial. Nevertheless, the frequency of follow-up may be decreased to improve practicality. While all enrolled patients completed baseline and follow up surveys, a significant proportion (~ 50%) did not complete a final fitness assessment and ~ 20% did not have follow up labs which may affect the ability to draw accurate conclusions from those data. In our analysis, we utilized the traditional (Ware) scoring of the SF36 in which improved scores on the scales within one domain (physical or mental) act to reduce the component score for the other domain which can affect outcomes and lead to bias. Newer approaches such as oblique rotation may be appropriate for future larger studies. Finally, the study was not designed nor powered to evaluate for specific cardiovascular outcomes which is of substantial interest in this patient population. The findings of the study cannot be generalized beyond this particular cohort in this particular geographic location.

## Conclusion

This prospective study evaluating a 6-month comprehensive CVD risk reduction program in patients with breast cancer receiving anthracycline and/or trastuzumab based therapy resulted in an improved patient-reported mental health, but a decline in patient reported physical health, despite improved or maintained markers of cardiovascular fitness, including a reduction in systolic and diastolic blood pressure. Incorporating similar programs in a real-world setting are likely to be both feasible and beneficial to patients. Moreover, this pilot data provides the foundation for future randomized controlled trials to evaluate the effects of a structured CVD risk reduction program in this high-risk cohort.

## Implications for practice

There is increasing recognition that breast cancer patients with cardiovascular risk factors are at higher risk for cardiotoxicity and future cardiovascular disease. An intervention that includes a motivational interviewing counseling style where the patient retains autonomy over their priorities for healthy behavior change is likely to be beneficial for developing intrinsic motivation for change. A comprehensive cardiovascular risk reduction program can improve patients’ perceived psychological well-being and may translate into long term cardiovascular health benefits for patients and survivors.

## Data Availability

The authors confirm that the data supporting the findings of this study are available within the article [and/or] its supplementary materials.
